# Beyond diagnosis in general practice: Predictive processing, a novel framework for persistent physical symptoms

**DOI:** 10.1080/13814788.2026.2717019

**Published:** 2026-08-18

**Authors:** Kees van Boven, Peter Lucassen, Tim C. Olde Hartman, Omer Van den Bergh

**Affiliations:** ^a^ Department of Primary and Community Care, Radboud University Nijmegen Medical Center, Nijmegen, The Netherlands; ^b^ Department of Health Psychology, University of Leuven, Leuven, Belgium

**Keywords:** Persistent physical symptoms, predictive processing, prior beliefs, symptom–test discordance, general practice, clinical explanation

## Abstract

**Background:**

Persistent physical symptoms are among the most common and challenging presentations in general practice, whether investigations are normal or reveal pathology insufficient to explain their severity. Patients and clinicians alike often experience these presentations as a dead end. Traditional biomedical models assume that symptoms passively reflect structural pathology, and that normal test results imply the absence of illness.

**Objectives:**

To introduce predictive processing as a theoretical framework for understanding persistent physical symptoms in general practice, and to outline its implications for clinical explanation and management.

**Methods:**

This opinion paper draws on the predictive processing and active inference literature to develop a clinically applicable account of symptom perception, illustrated with clinical scenarios from general practice.

**Results:**

Predictive processing proposes that symptoms are active perceptual constructions, generated by the brain as it infers the state of the body from incoming signals and prior beliefs. When prior beliefs of danger or irreversibility are strong, and peripheral signals are weak or ambiguous, these prior beliefs can dominate perception, producing substantial suffering in the absence of clear pathology. This framework explains symptom–test discordance and reframes normal results: they do not end clinical responsibility, but shift it towards modifying symptom-sustaining priors towards expectations of safety and recovery.

**Conclusion:**

For the general practitioner, this framework provides a coherent scientific rationale for explanation, continuity of care, expectation management, cognitive approaches and graded activity. These interventions emerge as core clinical tools, not consolation, when “nothing is found”.

## KEY MESSAGES

- Predictive processing explains why symptoms may persist despite normal or inconclusive clinical investigations.
- It accounts for discordance between reported symptoms and test results, and for variation between patients and over time.
- General practice interventions—expectation management, continuity of care, and graded activity—offer meaningful care beyond establishing a diagnosis.

## Persistent physical symptoms beyond diagnosis

A woman reports crushing fatigue three months after a viral infection. Blood tests are normal, imaging is reassuring, yet she cannot return to work [1]. Another patient, known with moderate knee osteoarthritis, rates his pain as severe and is unable to climb stairs, yet imaging shows only mild radiological changes [2]. A third, with moderate asthma, reports disabling breathlessness that correlates poorly with spirometry and varies with anxiety and context [3]. Such cases are familiar across specialties but are especially frequent in general practice, where the GP is typically the first and most continuous point of contact. They are not rare outliers but part of a large group of patients who experience substantial functional impairment with little or no correlating abnormality on standard investigations [1,4].

These patients are clearly suffering. The problem is not whether their symptoms are real, but how to understand them when diagnostic tests are normal. A clinical tradition emphasising structural pathology as the primary explanation for symptoms can leave clinicians with little to offer once investigations are unremarkable. Patients, for their part, may interpret normal results as evidence that their experiences are inexplicable, dismissed, or attributed entirely to psychological causes. The result is frustration on both sides and a persistent sense of being stuck in the consultation [1,4].

## How the brain constructs symptoms

Predictive processing is a theoretical framework for understanding how the brain generates perception, including bodily symptoms, by combining prior expectations with incoming sensory signals. It also helps explain why bodily symptoms do not always correspond to measurable pathology [5–7]. While this framework applies to symptom perception in general, its implications are most pressing where symptoms and investigations diverge. Rather than passively receiving signals from the body, the brain continuously generates predictions about bodily state, based on past experience and contextual cues. It compares these predictions with incoming sensory signals from nociceptors, interoceptors and other systems. Where signals confirm predictions, perception aligns with the model generating these predictions, and the process typically goes unnoticed. Where they diverge, ‘prediction errors’ arise and the brain updates its model of the body.

A central concept is the role of prior beliefs, hereafter: priors. These are learned, and in some cases innate, predictive models about the state of the body in health and illness, largely operating outside conscious awareness. We use the term ‘priors’ throughout. We reserve ‘beliefs’ for a proposition a patient could articulate, since much of what shapes symptom experience operates below that level. A person who has survived a myocardial infarction may develop a strong prior that chest discomfort signals imminent catastrophe. The same bodily signal that another person experiences as mild discomfort may be perceived as alarming in this context, not because the peripheral input differs, but because the interpretive framework does. Prior beliefs about danger, vulnerability and recovery all influence symptom experience [7,8].

In states such as post-infectious fatigue, persistent physical symptoms and functional disorders, this framework suggests that the brain may rely more heavily on priors than on peripheral signals. As a result, symptom intensity and quality may be shaped not only by measurable pathology, but also by expectations, illness beliefs, and the social and clinical context, including messages about prognosis and the meaning of symptoms [7]. This helps explain why some patients with extensive radiological osteoarthritis report little pain, while others with minimal changes are severely disabled, and why fatigue can persist long after inflammatory markers normalise [2,8].

This framework does not deny the role of structural pathology or peripheral input. Instead, it proposes that the relationship between structural pathology and symptoms varies along a continuum and depends on context, rather than being fixed. The same degree of structural pathology can produce very different symptom experiences in different patients, or in the same patient at different times [7,8]. Even when peripheral signals align closely with expectations, perception remains substantially shaped by priors, since symptom experience is the outcome of an inferential process rather than a direct readout of bodily state [8]. Symptoms are, in short, the brain’s best construction of bodily state given all available evidence, and that construction can persist and cause suffering even when investigations are reassuring.

## Why symptoms and tests diverge

Conventional investigations answer an important but limited question: ‘Is there measurable structural or functional abnormality in this tissue or organ?’ They cannot assess how the brain is weighting and interpreting incoming bodily signals. The result is a familiar clinical picture: severe symptoms with reassuring investigations, or, conversely, extensive structural pathology with relatively modest subjective complaints. This is seen in patients with advanced radiological osteoarthritis who report little functional impairment, where favourable priors appear to attenuate symptom perception [1,2,8]. This does not imply that peripheral input plays no role: perception is shaped by both peripheral and central processes. In osteoarthritis, for example, inflammatory processes not visible on imaging also influence how much weight nociceptive signals carry in the resulting perception.

From a predictive processing perspective, normal test results are not evidence that symptoms are trivial or imaginary. They show that a particular class of pathology is unlikely, but they say nothing about the inferential processes that generate symptoms. Normal results, or findings that do not sufficiently explain the severity of a patient’s symptoms, therefore shift the focus in clinical practice from searching for ever more subtle pathology to understanding and modifying symptom-sustaining priors towards expectations of safety and recovery [7,8].

## Explanation as a core intervention

This reconceptualisation has immediate implications for the general practice consultation. Explanation becomes a therapeutic act, not merely a way of delivering test results. A useful starting point is to summarise clearly what has and has not been found, and what has been actively ruled out. This can begin to update priors of danger: ‘We have looked carefully for heart disease that could explain your pain, and we have not found anything life threatening’. Crucially, clinicians should then check whether this has actually updated the patient’s beliefs. Asking ‘Does this make sense to you?’ or ‘Are there other worries that remain?’ ensures that reassurance is not merely delivered but received.

Explanation can then go further by introducing the idea that symptoms arise from how the nervous system interprets signals, especially after a period of illness and uncertainty [9,10]. For some patients the language of ‘brain processes’ is acceptable; others may respond better to metaphors such as a ‘sensitised alarm system’ or ‘symptom sensitivity’ that has become heightened. The crucial message is that this process is real, bodily and potentially reversible, not a sign that symptoms are imagined or fabricated [10,11].

Clinicians should anticipate that such explanations may initially be met with scepticism, especially if patients have previously encountered dismissive attributions that their symptoms were ‘all in the mind’. It is worth clarifying that predictive processing does not oppose brain and mind: thoughts, fears and expectations are themselves neural processes, and it is precisely through these that the brain constructs symptom experience. The message is not that symptoms are imaginary, but that understanding how the brain generates them opens the door to interventions that can change them [10].

## Eliciting priors and avoiding unhelpful investigation

If priors shape symptom experience, the patient is an indispensable informant about them. History-taking can extend beyond the traditional biomedical symptom inventory to include the patient’s own interpretations and context, exploring not only location, severity and timing, but also beliefs about causes, fears, previous illness and expectations of recovery: ‘What do you think is going on?’; ‘What worries you most?’; ‘What have previous doctors told you?’ These questions align with established frameworks such as ICE (Ideas, Concerns, Expectations) and SCEGS (Somatic, Cognitive, Emotional, General behavioural and Social factors). That they address cognitions, emotions and expectations is not incidental. Within the predictive processing framework, these psychological processes are themselves rooted in neural function; they shape how the brain constructs bodily experience and need updating if recovery is to occur [7,10,11].

When clinicians feel uncertain or pressured in the face of persistent symptoms and normal test results, the default response is often to repeat or extend investigations. From a predictive processing perspective this may be counterproductive: each additional test implicitly reinforces the prior that something serious may still have been missed, maintaining illness-sustaining beliefs in both patient and clinician [1,11]. Structured safety netting and planned follow-up, rather than open-ended diagnostic escalation, allow vigilance without inadvertently strengthening these priors [1,10].

## Working directly on prior beliefs

Many familiar interventions work directly on priors. Continuity of care through a stable, trusted relationship provides an ongoing contextual signal of safety [12]. Empathic, confident communication shapes expectations and perceived safety, modulating how bodily signals are interpreted [12,13]. Expectation management, framing recovery as likely and activity as safe, actively modifies priors towards safety; conversely, ambiguous or catastrophising communication can entrench beliefs of danger [11,13]. Cognitive behavioural approaches target maladaptive beliefs about symptoms and activity, helping patients revise predictions that amplify threat perception [11]. Graded activity and exposure provide lived experiences that disconfirm catastrophic priors: when feared activities are tolerated safely, prediction errors update expectations towards safety [11,14].

These interventions are not secondary options when ‘nothing is wrong’. Within a predictive processing framework, they are primary tools for altering the brain mechanisms that sustain symptoms. These interventions are effective in patients with or without structural pathology. We recognise that in many healthcare systems, time constraints and workforce pressures increasingly shift the management of persistent physical symptoms towards nurses, psychologists and other allied professionals. This does not diminish the relevance of the framework, since the same principles apply across disciplines. The GP’s distinctive contribution lies in continuity and in coordinating this care, whether delivered personally or through a multidisciplinary team. Table 1 summarises how this approach differs from a traditional model of care.

**Table 1.** Traditional and predictive-processing-informed approaches to persistent physical symptoms.

Dimension	Traditional approach	Predictive-processing-informed approach
Interpretation of normal results	Normal test results imply absence of illness; symptoms without findings are unexplained	Normal or non-explanatory results show that structural pathology is unlikely; the symptoms themselves remain real, generated by the brain’s construction of bodily state.
Role of investigation	Investigation continues or is repeated when symptoms persist despite earlier normal findings	Investigation is targeted and time-limited; further testing is used deliberately, not to reassure by default
The consultation	Focused on ruling pathology in or out; history-taking centres on location, severity, timing	Extends to eliciting priors: the patient’s ideas, fears, expectations, and past experiences with illness and healthcare
Communication	Normal results delivered as reassurance (“nothing is wrong”); explanation stops once pathology is excluded	Explanation continues as active treatment: what was found, what was ruled out, and how symptoms are generated and can change
Follow-up	Open-ended, often reactive; further escalation if symptoms persist	Structured safety-netting and planned review; continuity used deliberately to reinforce expectations of safety and recovery

## Education, policy and research

Current medical training devotes far more time to recognising pathology than to managing consultations in which no definitive diagnosis emerges [1,10,11]. Predictive processing explains how symptoms arise from the interaction between peripheral input and central processing, and why this relationship is sometimes close and sometimes weak or absent. It thereby provides a mechanistic foundation for the established biopsychosocial model, equipping trainees with tools to treat patients with persistent symptoms. At a systems level, relational continuity, adequate consultation time and multidisciplinary access are preconditions for this work [12]. Policies that prioritise diagnostic throughput over explanation and rehabilitation can undermine recovery-oriented care. Incentive structures and quality metrics should recognise the value of continuity, careful explanation and thoughtful non-investigation where appropriate.

Whether predictive-processing-informed consultation strategies improve outcomes, acceptability or consultation efficiency in primary care has not yet been formally evaluated. The framework currently offers an explanatory and communicative rationale rather than a tested intervention. Conceptually related structured communication interventions have nonetheless shown benefit in primary care: a Norwegian cluster-randomised trial of a work-focused communication tool, the Individual Challenge Inventory Tool, significantly reduced sick leave and improved patient-reported outcomes in patients with persistent physical symptoms [15]. Table 2 outlines priority questions for future research on predictive-processing-informed approaches specifically.

**Table 2.** Priority questions for future research on predictive-processing-informed care in general practice.

Domain	Research question
Training	How can predictive processing be taught effectively to general practitioners?
Patient acceptability	How acceptable are brain-based explanations to different patient groups?
Clinical outcomes	Does predictive-processing-informed communication improve patient understanding, reassurance, symptom outcomes, or healthcare utilisation compared with usual consultations?
Education and implementation	What educational strategies are needed for implementation across undergraduate, postgraduate and continuing GP education?

## Conclusion

Predictive processing offers a coherent account of why symptoms and tests so often diverge and why normal results do not end clinical responsibility. Symptoms are constructions, emerging from an inferential process in which priors, expectations and context interact with peripheral signals [5–7]. Persistent symptoms despite normal test results are thus not a failure of investigation or of patient credibility. They are an invitation to shift focus: from ‘What have we missed?’ to ‘What priors might be shaping this experience, and how can we help to update them?’

Continuity of care, honest explanation, expectation management and guided activity are not adjuncts to real treatment but core clinical tools. When deployed within an explicit predictive processing framework, they give longstanding ‘care beyond diagnosis’ a scientific foundation and make it teachable, in general practice and beyond [1,7,10,11].
